# Neglected intraocular wooden foreign bodies

**DOI:** 10.11604/pamj.2018.29.201.12092

**Published:** 2018-04-06

**Authors:** Hanane Oummad, amina Laghmari

**Affiliations:** 1 University of Mohamed V Souissi, Hôpital des, Spécialités, Ophtalmology A Department, Morocco

**Keywords:** Wooden foreign body, lens, iris

## Image in medicine

A 13-year-old child had left orbital trauma by wood. He consulted 2 week after for decreased visual acuity and eye pain. The ophthalmological examination showed the presence of a wound of autotight cornea a wooden foreign body incarcerated in the iris and the lens. Extraction of the foreign body associated with an adapted antibiotic treatment led to clinical improvement.

**Figure 1:**
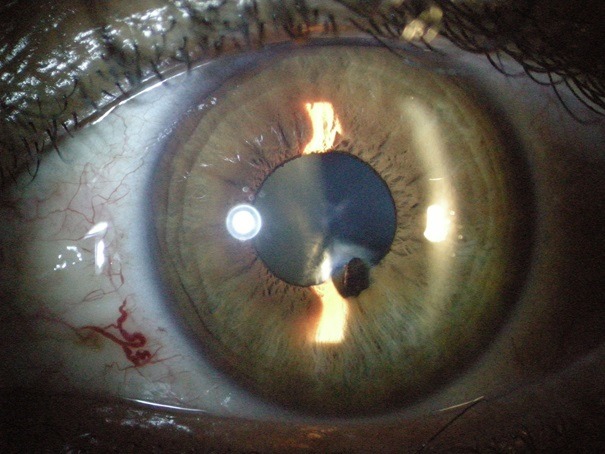
wooden foreign body incarcerated in the iris and the lens

